# Bamboo Leaf Flavonoids from *Phyllostachys glauca* McClure Suppress the Progression of Alzheimer’s Disease Induced by Circadian Rhythm Disruption Through Regulating Hif3α/Rab7/TNFα/IL1β Pathway

**DOI:** 10.3390/ijms26073169

**Published:** 2025-03-29

**Authors:** Junru Li, Victor I. K. Leung, Zixiang Xu, Taiyu Zhang, Jianing Du, Yuqing Zhang, Huiying Li

**Affiliations:** 1 Beijing Key Laboratory of Food Processing and Safety in Forestry, College of Biological Sciences and Technology, Beijing Forestry University, Beijing 100083, China; 18395270921@163.com (J.L.); xuzixiang25666@163.com (Z.X.); zhangtaiyu1120@163.com (T.Z.); djn15661258236@bjfu.edu.cn (J.D.); zyqingqing1128@163.com (Y.Z.); 2 Department of Metabolism, Digestion and Reproduction, Imperial College London, Du Cane Road, London W12 0NN, UK; v.leung23@imperial.ac.uk

**Keywords:** circadian rhythm disruption, Alzheimer’s disease, neuroinflammation, hypoxia-inducible factor 3α, bamboo leaf flavonoids

## Abstract

Circadian rhythm disruption is a modifiable risk factor for Alzheimer’s disease (AD) progression, marked by neuroinflammation, oxidative stress, and amyloid-β (Aβ) accumulation. Hypoxia-inducible factor 3α (Hif3α) has emerged as a key regulator of inflammatory and oxidative pathways. To evaluate the impacts of circadian disruption on AD progression and investigate the therapeutic potential of bamboo leaf flavonoids (BLFs), C57BL/6N mice (normal mice) and APP/PS1 transgenic mice (AD mice) were exposed to circadian disruption via randomized light exposure and stress, as the in vivo model. Then, BLFs were administered to assess effects on neuroinflammation, oxidative stress, and organ damage. Next, Nissl body staining and Aβ protein immunohistochemistry were performed to evaluate the effects of BLFs on brain pathology. Through transcriptome sequencing, key factors and the related pathway were screened out. In vitro, molecular mechanisms were explored in PC12 cells treated with Aβ42 and *Hif3α* siRNA fragments. Results demonstrated that circadian disruption increased oxidative stress and early liver and kidney damage degrees, with greater severity in AD mice. BLFs partially reversed oxidative damage and reduced Aβ deposition. Transcriptome analysis revealed upregulation of *Hif3α* in circadian-disrupted mice, linked to inflammation and oxidative stress. In vitro, the knockdown of *Hif3α* reduced inflammation and normalized protein expression, which could be regulated by BLFs and suppressed AD progression. In conclusion, circadian disruption exacerbated AD progression via regulating Hif3α/Rab7/TNFα/IL1β pathway. BLFs offered neuroprotection roles by mitigating inflammation and oxidative damage, highlighting Hif3α as a promising target for AD therapy and biomarker development.

## 1. Introduction

Alzheimer’s disease (AD), the most prevalent cause of dementia, affects approximately 46.8 million people globally—a number projected to rise to 131.5 million by 2050 [1]. Clinically, AD presents with progressive cognitive decline, memory loss, and behavioral changes, ultimately leading to death. While available treatments offer temporary symptom relief, there remains no cure for AD [2]. AD is characterized by two primary biomarkers: the deposition of beta-amyloid proteins (Aβ) and the entanglement of tau proteins. Aβ, in particular, serves as a major pathophysiological marker and therapeutic target in AD [3,4]. Beyond these molecular markers, patients frequently experience sleep disturbances and circadian rhythm disruptions that may exacerbate AD progression [5].

Circadian rhythms regulate essential functions like sleep–wake cycles, hormone release, and metabolism, driven by the central biological clock located in the hypothalamus, known as the suprachiasmatic nucleus (SCN). The SCN synchronizes peripheral clocks across organs and cells, facilitated by light input via the retina and signals from the autonomic nervous and the hypothalamo-pituitary system, involving neurotransmitters such as melatonin and cortisol [6]. Peripheral clocks respond mainly to factors like diet and physical activity, termed “zeitgebers”, which modulate SCN timing. Misalignment between central and peripheral clocks disrupts circadian rhythms and impairs neurophysiological processes, with circadian rhythm disruption increasingly recognized as a risk factor for AD [7,8].

Recent evidence suggests that hypoxia-inducible factor 3α (Hif3α), part of the HIF family of transcription factors, may play a critical role in this process. Hif3α regulates genes involved in inflammation and oxidative stress and can be activated independently of oxygen levels by inflammatory stimuli, linking it to various pathological conditions [9]. Studies found that Hif3α might play key roles in neurodegenerative diseases, including diabetic cognitive impairment and AD-like phenotype in gene knockdown models [10,11]. However, the particular molecular mechanism related to Hif3α’s role in AD and other neurodegenerative diseases remains underexplored.

Given the obvious limitations of current approaches for AD, including unclear mechanisms, undefined targets, and possible side effects, people have begun to consider traditional Chinese medicines with low toxicity, multi-target, and multi-effect properties as a promising alternative source of bioactive compounds. Bamboo leaf flavonoids (BLFs), derived from bamboo (*Phyllostachys glauca* McClure) leaf extracts, comprise a complex of four bioactive compounds: orientin (OT), homoorientin, vitexin (VX), and isovitexin (IsVX) [12]. These compounds, both individually and collectively, have demonstrated therapeutic properties, including antioxidant, anti-cancer, anti-inflammatory, anti-hyperalgesic, and neuroprotective effects [13,14,15,16]. Additionally, some flavonoids within BLFs, such as OT and VX, have been suggested to exert therapeutic effects, thereby improving health outcomes [17]. Given that inflammation is implicated in the pathophysiology of AD, BLFs may play a role in arresting disease progression and alleviating symptoms. It has recently been suggested that flavonoids have pharmacological effect on AD [18]. Previous animal and human studies suggest that polyphenols and flavonoids derived from natural extracts may provide therapeutic benefits in treating AD [19,20]. Due to their broad range of bioactive effects, including the modulation of inflammatory pathways that involve Hif3α, BLFs warrant further investigation as a potential treatment for AD.

Although there is increasing evidence linking circadian disruption to the pathogenesis of AD, the exact mechanisms by which circadian misalignment exacerbates AD remain incompletely understood. In particular, the role of neuroinflammation, involving pathways such as Hif3α that are influenced by disrupted circadian rhythms, in the progression of AD requires further elucidation. Current therapeutic strategies for AD primarily focus on symptomatic management, highlighting the need for interventions targeting underlying pathophysiological processes, including inflammation and oxidative stress. Given the anti-inflammatory and neuroprotective properties of BLFs, along with emerging evidence implicating Hif3α in inflammatory responses, BLFs present a promising therapeutic approach.

In the present study, we hypothesized that circadian rhythm disruption accelerated AD pathology through Hif3α-mediated inflammation and oxidative stress and that BLFs might attenuate these effects. This study aimed to evaluate the capacity of BLFs to mitigate circadian disruption’s impact on AD, contributing to the development of novel disease-modifying therapies. By constructing an AD progression model induced by circadian rhythm disruption in vivo and an Aβ42-stimulated cell model in vitro, we evaluated the efficacy of BLFs in alleviating neuron damage. Further investigation revealed that the underlying molecular mechanism is regulated by Hif3α/Rab7/TNFα/IL1β pathway.

## 2. Results

### 2.1. Brain Index and Biochemical Indicators in Mouse Serum

The Brain Index (brain weight/body weight × 100%, %) was used to assess the impact of circadian disruption and BLFs treatment on normal and AD mice (Figure S1B). Circadian disruption did not significantly affect the Brain Index in either cohort. However, AD mice exhibited lower Brain Index values compared to normal mice in both control and BLFs-treated groups (*p* < 0.001). BLFs treatment did not significantly improve the Brain Index in AD mice (*p* = 0.68), nor did it significantly alter the Brain Index in normal mice (*p* = 0.91 and *p* > 0.99 for C+D and M+D vs. controls). These results suggest that AD pathology, rather than circadian disruption, drives the reduction in Brain Index, with no measurable effect from BLFs treatment.

Circadian disruption consistently elevated markers of oxidative stress and organ damage in both normal and AD mice (Figure S1C). MDA levels, an indicator of oxidative stress, were significantly increased in the circadian disruption groups (M) (*p* < 0.001), while GSH, an endogenous antioxidant, was significantly depleted (*p* < 0.001), indicating a marked oxidative imbalance caused by disrupted circadian rhythms. In addition to oxidative stress, circadian disruption resulted in significant liver and kidney dysfunction. Elevated levels of ALT and AST were observed in both cohorts, indicating liver damage (*p* < 0.001), while markers of renal impairment, Cr and BUN, were similarly elevated (*p* < 0.001).

BLFs treatment (M+D) partially reversed the effects of circadian disruption on oxidative stress and organ damage (Figure S1C). In both normal and AD mice, MDA levels were significantly reduced and GSH levels significantly increased following BLFs treatment. Similarly, liver and renal dysfunction markers (ALT, AST, BUN, and Cr) improved significantly in the BLFs-treated groups (*p* < 0.001). However, BLFs treatment did not appear to improve oxidative stress or organ damage markers in AD mice without circadian disruption (*p* > 0.05).

### 2.2. Histopathological Observation of Liver and Kidney Tissue

**Figure 1:**
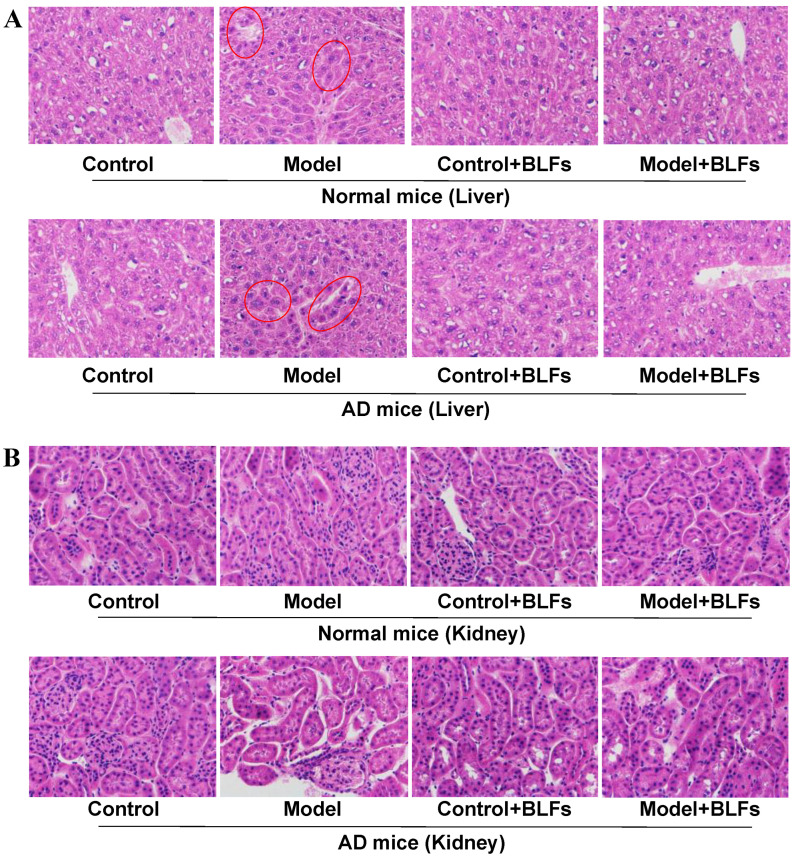
HE staining results of liver/kidney tissue. (**A**) HE staining of liver tissue in normal and AD mice across treatment groups (Control, Model, Control+BLFs, and Model+BLFs). In normal mice, mild inflammation is observed in the Model group, while AD mice under circadian disruption show hepatocyte disorganization and swelling (circled). BLFs treatment partially reduced liver damage in both cohorts. (**B**) HE staining of kidney tissue shows preserved architecture in normal mice across all groups. In AD mice, circadian disruption caused glomerular damage and tubular dilation (circled), which was partially improved with BLFs treatment. n = 3.

Histological analysis of the liver tissue (Figure 1A) revealed mild inflammation in the Model group of normal mice, whereas AD mice subjected to circadian disruption exhibited more pronounced liver damage, including hepatocyte disorganization and cellular swelling. BLFs treatment partially reduced these pathological changes but did not fully restore normal liver architecture in either cohort. In the kidney (Figure 1B), circadian disruption led to minor changes in normal mice, whereas AD mice showed significant glomerular damage and tubular dilation. BLFs treatment resulted in some improvement in both liver and kidney pathology, although damage remained evident, particularly in AD mice.

### 2.3. Staining of Special Markers in Brain Tissue: Nissl Bodies and Amyloid-β (Aβ) Protein

**Figure 2:**
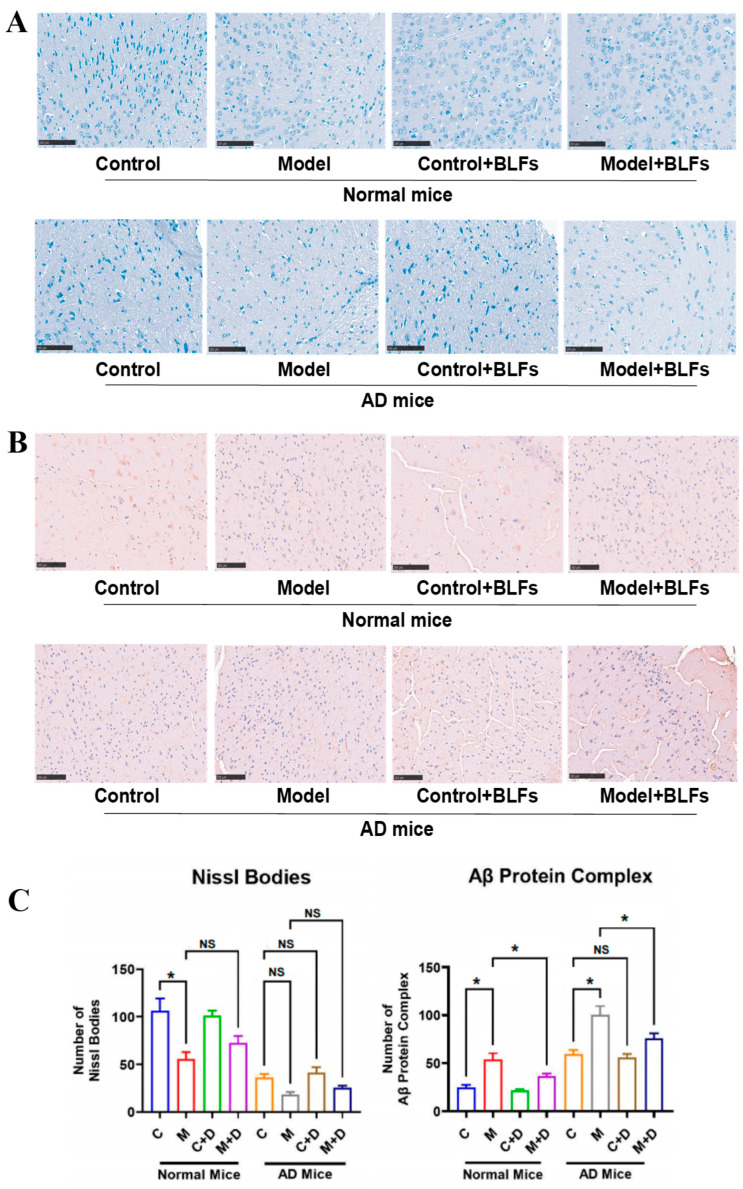
Nissl body and Aβ protein staining of brain tissue. (**A**) Toluidine blue staining of Nissl bodies in normal and AD mice across four experimental groups (Control, Model, Control+BLFs, and Model+BLFs). Circadian disruption caused significant reductions in Nissl body numbers in normal mice, with fragmented Nissl bodies in AD mice. BLFs treatment did not significantly restore Nissl body numbers in either cohort. (**B**) Immunohistochemical staining of amyloid-beta (Aβ) proteins in brain tissue. Circadian disruption increased Aβ deposition in both cohorts, with AD mice showing more extensive accumulation. BLFs treatment significantly reduced Aβ deposits, although deposition remained higher in AD mice. (**C**) Quantification of Nissl bodies and Aβ protein complexes in normal and AD mice. Statistical significance (*p*-values) is indicated for each comparison. n = 3. * indicates *p* < 0.05; NS indicates no significant difference (*p* ≥ 0.05). C stands for the control group without any treatment, M stands for circadian disruption treatment, D stands for BLFs treatment.

Toluidine blue staining revealed a significant reduction in Nissl bodies in normal mice following circadian disruption (Model) (*p* < 0.001), while the decrease in AD mice was not statistically significant (*p* = 0.09) (Figure 2A). In AD mice, circadian disruption led to fragmented and disorganized Nissl bodies, indicative of neuronal stress. BLFs treatment (Control+BLFs and Model+BLFs) showed no significant recovery in Nissl body numbers in either cohort (*p* > 0.05), suggesting that BLFs did not substantially restore neuronal integrity.

Immunohistochemical staining for Aβ protein revealed significant deposition in both normal and AD mice subjected to circadian disruption (Model) (*p* < 0.001) (Figure 2B). AD mice exhibited extensive Aβ aggregation following circadian disruption. BLFs treatment resulted in a significant reduction in Aβ deposits in both cohorts (*p* < 0.001), although Aβ accumulation remained higher in AD mice compared to normal mice. These results suggest that BLFs treatment reduces amyloid deposition, independent of circadian disruption or AD pathology.

### 2.4. Astrocyte and Microglia Activation in Brain Tissue: GFAP/Iba-1 Double Staining

**Figure 3:**
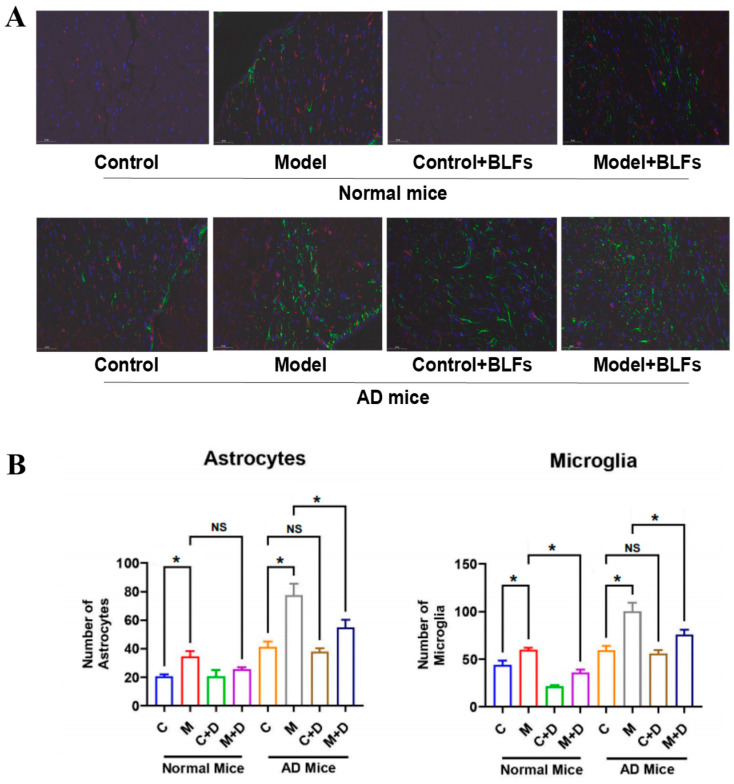
GFAP/Iba-1 fluorescence double staining of brain tissue. (**A**) Dual immunofluorescence staining of astrocytes (GFAP, green) and microglia (Iba1, red) in brain tissue of normal and AD mice under control, circadian disruption (Model), and BLFs-treated conditions. Circadian disruption led to increased astrocyte and microglial activation in both cohorts. BLFs treatment reduced activation. (**B**) Quantification of astrocytes and microglia in normal and AD mice. Circadian disruption significantly increased the number of astrocytes and microglia in both cohorts (*p* < 0.05 in normal mice, *p* < 0.001 in AD mice), and BLFs treatment partially mitigated these effects (*p* < 0.001). n = 3. * indicates *p* < 0.05; NS indicates no significant difference (*p* ≥ 0.05). C stands for the control group without any treatment, M stands for circadian disruption treatment, D stands for BLFs treatment.

Dual immunofluorescence staining using GFAP and Iba1 revealed increased activation of astrocytes and microglia across all experimental groups (Figure 3A). In the AD Model group, astrocyte activation, indicated by stronger GFAP staining (green), was more prominent, while microglia activation (red) was elevated across both normal and AD cohorts. Circadian disruption significantly increased the number of astrocytes and microglia in both normal (*p* < 0.05) and AD mice (*p* < 0.001) compared to controls (Figure 3B). BLFs treatment resulted in a visible reduction in both astrocyte and microglial activation in normal and AD mice, though levels remained higher than those in the controls, particularly in AD mice. These findings suggest that BLFs partially attenuate the neuroinflammatory response induced by circadian disruption (*p* < 0.001).

### 2.5. Transcriptional Analysis of Brain Tissue in Response to Circadian Disruption and BLFs Treatment

**Figure 4:**
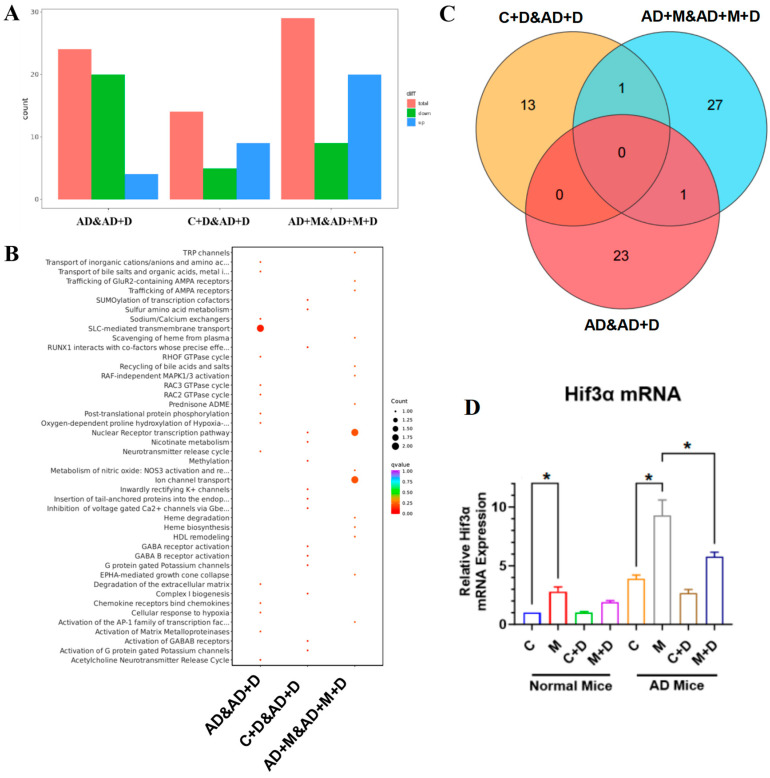
Investigation and analysis of transcription differentials in brain tissue. (**A**) Bar graph summarizing the total number of differentially expressed genes across experimental conditions (AD, BLFs-treated, circadian disruption, and control groups). (**B**) Gene Ontology (GO) enrichment analysis showing biological processes associated with differentially expressed genes. Larger dots indicate a stronger relevance to processes such as oxidative stress, inflammation, and synaptic function. (**C**) Venn diagram illustrating the overlap and unique transcriptional changes across treatment groups, highlighting shared and distinct gene expression patterns in normal and AD mice. (**D**) Quantitative analysis of *Hif3α* mRNA expression, demonstrating significant upregulation following circadian disruption in both normal and AD mice, with BLFs treatment reducing *Hif3α* mRNA levels, particularly in AD mice. n = 5. * indicates *p* < 0.05. C stands for normal mice, AD stands for AD mice, M stands for circadian disruption treatment, D stands for BLFs treatment.

To investigate transcriptional differences in response to circadian disruption and BLFs treatment in normal and AD mice, we conducted a comprehensive transcriptome analysis of brain tissue across all experimental groups (Figure 4). The analysis revealed distinct transcriptional profiles among the groups, with differential gene expression patterns associated with both circadian disruption and AD pathology.

The bar graph (Figure 4A) summarizes the total number of differentially expressed genes across different conditions, highlighting the transcriptional impact of BLFs treatment. Gene Ontology (GO) enrichment analysis (Figure 4B) revealed significant biological processes impacted by these differentially expressed genes, including pathways related to oxidative stress, inflammation, and synaptic function. Larger dots in the enrichment plot correspond to higher relevance to specific biological processes, with BLFs treatment mitigating gene expression changes associated with these processes.

The Venn diagram (Figure 4C) displays the overlap and unique transcriptional changes across different treatment groups. Notably, certain transcriptional changes were specific to the AD cohort, while others were shared between normal and AD mice. Finally, the quantitative analysis of *Hif3α* mRNA expression (Figure 4D) showed significant upregulation following circadian disruption, with BLFs treatment reducing *Hif3α* mRNA levels in both cohorts, although this effect was more pronounced in AD mice (*p* < 0.001).

### 2.6. Expression of the Hif3α/Rab7/TNFα/IL-1β Pathway in Mouse Brain Tissue

**Figure 5:**
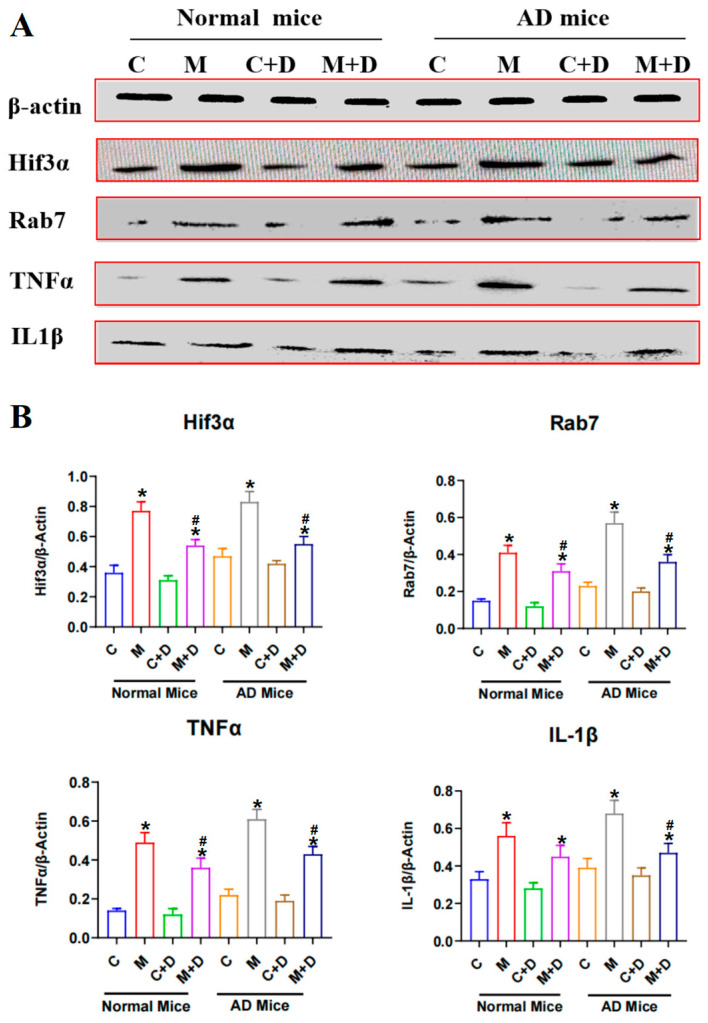
Expression of Hif3α/Rab7/TNFα/IL1β pathway in mouse brain tissue. (**A**) Western blot analysis of Hif3α, Rab7, TNFα, and IL-1β protein expression in brain tissue of normal and AD mice across four experimental conditions (Control, Model, Control+BLFs, and Model+BLFs). Circadian disruption significantly increased the expression of all four proteins, particularly in the AD cohort. (**B**) Quantification of Hif3α, Rab7, TNFα, and IL-1β relative to β-actin. BLFs treatment significantly reduced TNFα and IL-1β levels in both normal and AD mice, with a more pronounced effect in the AD cohort. Rab7 and Hif3α levels were similarly reduced following BLFs treatment in circadian-disrupted groups, though their expression remained unaffected in AD mice without circadian disruption. n = 5. C stands for the control group, M stands for circadian disruption treatment, D stands for BLFs treatment. * compared with the control group (C), *p* < 0.05; # compared with the model group (M), *p* < 0.05.

Levels of inflammatory cytokines TNFα and IL-1β were significantly elevated in both normal and AD mice following circadian disruption, with AD mice showing a more marked rise (Figure 5). This indicates that AD pathology exacerbates the neuroinflammatory response triggered by circadian rhythm disturbances. BLFs treatment effectively reduced levels of TNFα and IL-1β in treated groups, with AD mice, experiencing notable reductions, indicating potential protection against inflammation-induced neuronal damage. Circadian disruption also led to elevated levels of Rab7 and Hif3α in both normal and AD mice, with higher expression in the AD cohort, suggesting a compounded effect of circadian disruption on AD pathology. While BLFs treatment reduced Rab7 and Hif3α levels following circadian disruption in both cohorts, it did not significantly alter their expression in AD mice without circadian disruption, suggesting that BLFs may primarily alleviate circadian disruption stress rather than AD pathology alone.

### 2.7. Expression of the Hif3α/Rab7/TNFα/IL-1β Pathway in PC12 Cells

**Figure 6:**
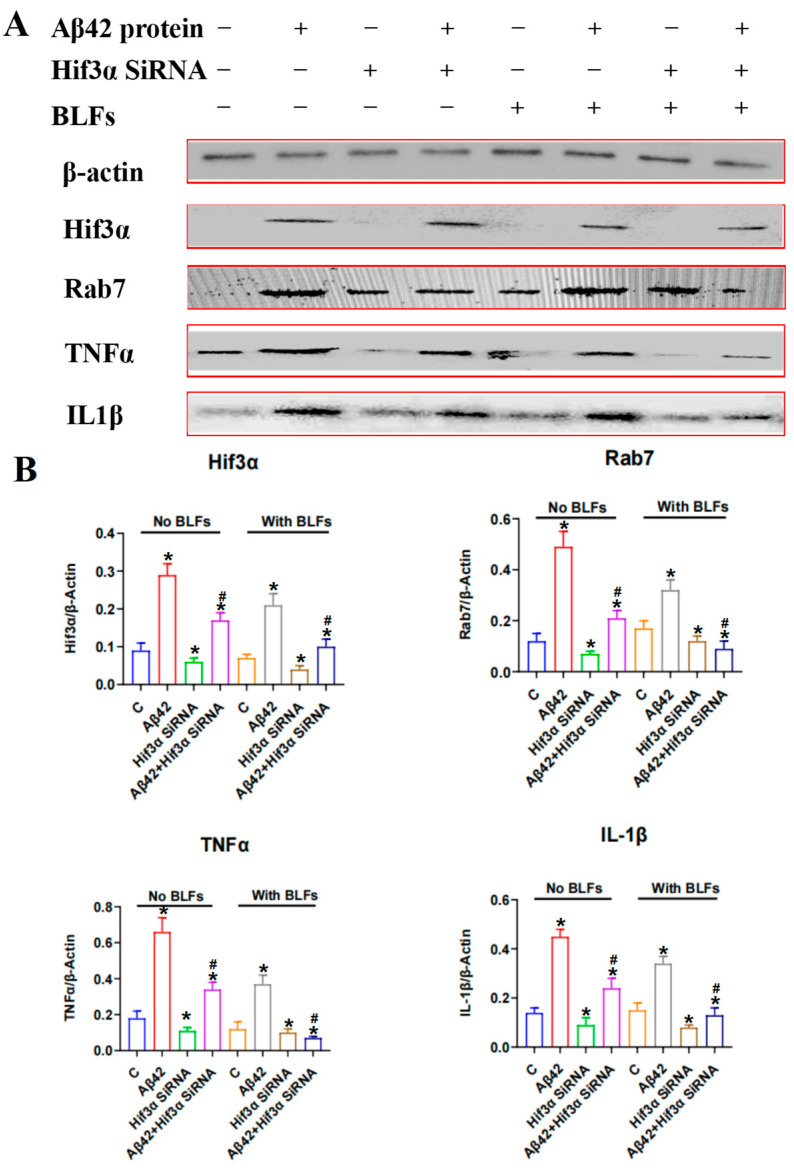
Expression of Hif3α/Rab7/TNFα/IL1β pathway in PC12 cells. (**A**) Western blot analysis of *Hif3α*, Rab7, TNFα, and IL-1β in PC12 cells subjected to Amyloid-beta (Aβ42) treatment, *Hif3α* knockdown (siRNA), and BLFs treatment. Aβ42 treatment significantly increased the expression of all proteins, indicating elevated inflammation and cellular stress. (**B**) Quantification of protein expression relative to β-actin. *Hif3α* knockdown, with or without BLFs, was the most effective intervention in reducing the expression of Hif3α, Rab7, TNFα, and IL-1β. BLFs treatment alone partially mitigated the effects of Aβ42, reducing both inflammatory and aberrant protein levels. n = 3. * compared with the control group (C), *p* < 0.05; # compared with the Aβ42 group, *p* < 0.05.

PC12 cells were subjected to Amyloid-beta (Aβ42), *Hif3α* knockdown (siRNA), and BLFs, with protein expression analyzed via Western blot (Figure 6). Aβ42 treatment markedly increased the levels of inflammatory markers (TNFα and IL-1β) and elevated the expression of aberrant proteins, including Hif3α and Rab7. Among the interventions tested, *Hif3α* knockdown, with or without BLFs, most effectively suppressed inflammation and normalized protein expression. In the absence of *Hif3α* knockdown, BLFs treatment alone partially reduced the expression of inflammation markers and aberrant proteins.

## 3. Discussion

In this study, we investigated the effects of circadian disruption on oxidative stress, neuroinflammation, and AD pathology in normal and AD-prone mice, and explored the therapeutic potential of BLFs and *Hif3α* knockdown, using in vivo and in vitro models. Numerous studies have shown that circadian rhythm disruption can lead to oxidative stress and organ damage [7,21]. Our findings also indicate that circadian rhythm disruption results in increased oxidative stress and early liver and kidney damage. Notably, the extent of liver and kidney damage was more pronounced in Alzheimer’s disease (AD) mice compared to normal mice. Biochemical indicators revealed a decrease in GSH levels and an increase in MDA, ALT, and AST levels, indicating elevated oxidative stress. Histopathological examination showed mild liver inflammation in normal mice, while AD mice displayed severe hepatic cellular disarray and more evident kidney damage. Additionally, we found that BLFs treatment mitigated the oxidative stress and structural damage to the liver and kidneys caused by circadian rhythm disruption; however, significant damage remained in AD mice, suggesting that BLFs have limited therapeutic efficacy in the context of AD.

Through immunospecific staining, we observed that in AD mice, the key pathological marker of Alzheimer’s disease, Nissl bodies [22,23], appeared fragmented, disorganized, and reduced in number, with increased Aβ protein accumulation. Under circadian rhythm disruption, Nissl bodies were further reduced (*p* > 0.05), suggesting that circadian rhythm disruption may exacerbate AD symptoms to some extent. Additionally, the astrocyte marker GFAP [24] and the microglial marker Iba-1 [25] were elevated in AD mice, with hypertrophy of astrocytes and activation of microglia observed in both normal and AD mice following circadian rhythm disruption. This indicates that circadian rhythm disruption induced neuroinflammation, thereby aggravating the progression of AD [26]. Furthermore, we found that BLFs treatment significantly reduced Aβ protein deposition in both normal and AD mice, and markedly decreased the number of activated microglia in circadian rhythm-disrupted normal and AD mice. However, in AD mice, BLFs treatment did not significantly restore the integrity of Nissl bodies.

Previous studies have indicated that circadian rhythm disruption affects the expression of the HIF signaling pathway [27]. Similarly, our transcriptome sequencing results showed a significant increase in *Hif3α* mRNA levels in both normal and Alzheimer’s disease (AD) mice following circadian rhythm disruption (*p* < 0.001). This increase is associated with cognitive impairment, blood-brain barrier dysfunction, and the NF-κB inflammatory pathway [28]. However, current research on Hif3α’s role in brain health primarily focuses on stroke rather than neurodegenerative diseases [29]. Therefore, we further explored the mechanistic role of Hif3α in AD under circadian rhythm disruption. In mouse brain tissue, we found that circadian rhythm disruption significantly elevated the expression levels of Rab7, Hif3α, and inflammatory cytokines TNFα and IL-1β. BLFs treatment effectively reduced TNFα and IL-1β levels in both normal and AD mice, although it had no significant impact on Rab7 and Hif3α, especially in AD mice not affected by circadian rhythm disruption. Rab7, a small GTPase, has been implicated in autophagy processes, which may be impaired under circadian rhythm disruption—a process crucially involved in AD pathology [26]. Additionally, the hypoxia-inducible factor Hif3α interacts with pro-inflammatory cytokines TNFα and IL-1β, creating a feedback loop in inflammatory signaling [30]. To further identify potential therapeutic targets, we conducted corresponding in vitro experiments in PC12 cells (a rat adrenal pheochromocytoma cell line). The results showed that Aβ42 treatment significantly increased the levels of inflammatory markers (TNFα, IL-1β) and abnormal proteins (Hif3α, Rab7), which aligned with our findings in mouse brain tissue. In PC12 cells, *Hif3α* gene knockdown, with or without BLFs, was the most effective approach for reducing inflammation and normalizing protein expression, while BLFs alone only partially alleviated the effects of Aβ42. Unlike Rab7, whose upregulation is more likely a marker of disease progression than a therapeutic target, Hif3α actively participates in molecular pathways driving neuroinflammation, making it a potential target for neurodegeneration treatment. For the reasons of limited human relevance due to reliance on animal models, as well as the lack of long-term treatment data, the effects of BLFs in suppressing AD or other neurodegenerative diseases have not been fully elucidated. Moreover, the progression of these diseases induced by circadian rhythm disruption remains unclear. In addition, the potential variability in BLFs composition also required further investigation, which is essential for the clinical development of BLFs.

## 4. Methods

### 4.1. Reagents

The main materials and reagents used in this study are listed in Table S1. The space distributions of *Phyllostachys glauca* McClure in China are demonstrated in Figure S2. Bamboo leaf flavonoids (BLFs) complex extracted from *Phyllostachys glauca McClure* was purchased from J&K Scientific Ltd. (Beijing, China).

### 4.2. Animal Models

The animal experiments in this study were approved by the Ethics Committee of the Sinoresearch Biotechnology Co., Ltd. (Beijing, China; permission number: ZYZC20230506S). C57BL/6N mice (wild-type) and APP/PS1 mice (Alzheimer’s disease [AD] model) were randomly divided into four groups each (eight groups in total, n = 5 per group): (1) normal control group (no circadian disruption [CD] and no treatment); (2) CD group (subjected to CD with no treatment); (3) BLF control group (no CD, treated with BLF); and (4) CD with BLF group (subjected to CD and treated with BLF).

Mice not subjected to CD were maintained on a 12h light/12h dark cycle (light: 08:00–20:00; dark: 20:00–08:00). CD groups were housed in chambers covered with dark, opaque cloth for 24 h, with lighting randomly manipulated to switch on and off in an irregular pattern. In addition, CD groups were exposed to random sound disturbances using recordings of dog barking. Figure S1A presents a schematic diagram of the CD protocol. All other housing conditions were consistent across groups, and interventions continued uniformly for four weeks.

Mice in the BLFs treatment groups received oral gavage of BLFs at 0.1 g/kg body weight once daily, while control groups received an equivalent volume of sterile physiological saline. On day 29, all mice were euthanized via cervical dislocation. Blood samples and organs were collected for further analysis.

### 4.3. Detection of Biochemical Indicators

Serum samples (0.6 mL per sample) were diluted four-fold before being analyzed for biochemical markers using an automatic biochemical analyzer (Mindray BS-800, Mindray Bio-Medical Electronics Co., Ltd., Shenzhen, China). Biochemical indicators related with organ functions included alanine aminotransferase (ALT), aspartate transaminase (AST), creatinine (Cr), blood urea nitrogen (BUN), reduced glutathione (GSH), and malondialdehyde (MDA), according to the protocols of ELISA kits. n = 5 per group.

### 4.4. HE Staining of Liver and Kidney

Liver and kidney tissues were excised and weighed (n = 5 per group), organ indices were calculated as organ weight (g) × 100%/body weight (g). Tissue samples (5 × 5 × 2 mm) from the liver and kidney tissues were fixed in 10% formalin for 24 h, embedded in paraffin, and sectioned using a microtome (Leica, Wetzlar, Germany), followed by incubation at 40 °C for 12 h. A portion of the brain tissue (about 0.5 g) was frozen in liquid nitrogen and stored at −80 °C for subsequent transcriptomic analysis. Histological sections of the liver and kidney were stained with hematoxylin-eosin (HE) and examined under an optical microscope (Nikon, Tokyo, Japan) equipped with a camera (Olympus, Center Valley, USA). Blood samples (1 mL per mouse) were collected from the posterior orbital plexus, with half (0.5 mL) placed in heparinized tubes for hematological analysis.

### 4.5. Special Factor Staining of Brain Tissues

For Nissl staining, brain sections (6–8 μm) were dewaxed, rehydrated, and stained in 1% toluidine blue for 40 min, followed by distilled water rinsing, graded alcohol dehydration, xylene clearing, and neutral balsam sealing for microscopic observation. For Aβ staining, paraffin-embedded brain sections were deparaffinized, rehydrated, and subjected to antigen retrieval with 0.01 mol/L sodium citrate buffer. Sections were treated with 3.0% hydrogen peroxide to block peroxidase activity, followed by a 1h blocking step. Primary Aβ antibody was applied to treat the sections overnight at 4 °C, followed by incubation with secondary antibody for 1 h and color development. n = 5 per group.

For GFAP/Iba-1 double staining, brain sections were blocked in 2% BSA/1.5% Triton/PBS, then incubated overnight at 4 °C with primary antibodies against GFAP (astrocytes) and Iba-1 (microglia) at 1:1000. After PBS washing, sections were incubated with secondary antibodies for 1 h at room temperature. Nuclei were stained with DAPI, and fluorescence images were captured using an FV10i microscope (Olympus, Japan) with appropriate filters for DAPI (nuclei), Alexa Fluor 488 (GFAP), and Alexa Fluor 594 (Iba-1). n = 5 per group.

### 4.6. Transcriptome Sequencing and Analysis of Brain Tissue

Transcriptome sequencing was outsourced to Wuhan MetWare Biotechnology Inc. (Wuhan, China). Eight brain tissue samples were randomly selected from each group: normal control, normal CD (no treatment), normal (no CD with treatment), normal CD (with treatment), AD control, AD (no CD with treatment), AD CD (no treatment), and AD CD (with treatment). mRNA with polyA tails was isolated from total RNA using Oligo(dT) magnetic beads (Solarbio).

The mRNA fragments served as templates for cDNA synthesis, which was purified, end-repaired, A-tailed, and ligated with sequencing adapters. PCR amplification generates cDNA libraries, which were pooled and sequenced using the Illumina platform.

Raw data were processed using fastp software (v0.23.4) to remove low-quality reads (Q ≤ 20), reads containing sequencing adapters, and paired reads with more than 10% unidentified bases (N). Sequencing error rates and GC content distributions were evaluated to ensure data quality, and the final sequences were obtained.

Data were processed using fastp (v0.23.4) to remove low-quality reads (Q ≤ 20), sequencing adapters, and paired reads with >10% unidentified bases. DESeq was employed to identify differentially expressed genes, with gene counts computed using feature counts. Visualization included statistical plots, volcano plots, heatmaps, Venn diagrams, and scatter plots for gene ontology (GO) enrichment.

### 4.7. PC12 Cells Culturing and siRNA Fragment Transfection

Frozen PC12 cells were rapidly melted in a 37 °C water bath and centrifuged at 1200× *g* rpm for 5 min, the cell pellet was collected. The cells were resuspended in 10 mL of complete medium (2.5% FBS + 5% HS + 91.5% DMEM + 1% Penicillin-Streptomycin), then the cell suspension was transferred into a culture dish, and incubated in a CO_2_ incubator. After the cells adhered and grew for about 6 h, 50 ng/mL NGF was added into the wells to induce differentiation. Over 4–6 days’ observation, the cells were treated with Aβ42 (15 μM) to simulate an Alzheimer’s pathological environment, while others were treated with BLFs (1.5 g/L). Additionally, certain cells (about 10^5^ per well) were transfected with siRNA fragment (0.1 μM) to knock down *Hif3α* expression. The transfection method was performed using a lipofectamine 2000 kit according to the manufacturer’s instructions. n = 3 per group.

### 4.8. Western Blot Detection of Special Proteins

Protein levels of Hif3α, Rab7, TNFα, and IL-1β in brain tissue and PC12 cell were assessed by Western blot. A total of 0.3 g of tissue sample in each mouse was lysed using RIPA buffer containing phenylmethylsulfonyl fluoride (PMSF). Ten-microliter samples and a pre-stained protein marker were electrophoresed on SDS-PAGE at 120 V for 30 min, and 130 V for 40 min. Proteins were transferred onto polyvinylidene fluoride membranes, blocked in 5% skimmed milk, and incubated overnight at 4 °C with primary antibodies. After washing for three times (5 min × 3), membranes were treated with horseradish peroxidase-conjugated secondary antibodies (1:3000 dilution) for 2 h, followed by enhanced chemiluminescence (ECL) detection. Protein expression levels were quantified relative to β-actin using a Monad imaging system. n = 3 per group.

### 4.9. Statistical Analysis

Data are presented as mean ± standard deviation (SD). Statistical analyses were performed using GraphPad Prism version 10.1.2. Group comparisons were performed with *t*-tests, with statistical significance set at *p* < 0.05.

## 5. Conclusions

This study demonstrates that circadian disruption exacerbates neuroinflammation, oxidative stress, and organ damage in Alzheimer’s disease, while *Hif3α* inhibition and BLFs offer partial protection. Our findings underscore Hif3α’s role in AD progression, and suggest shared risk pathways with stroke, emphasizing Hif3α as a potential therapeutic target. Future research should further explore the links between circadian disruption, Hif3α activity, neuroinflammation, and autophagy, offering new avenues for AD treatment strategies.

## Data Availability

All data generated and analyzed to support the findings of this study are included within the article and Supplementary Materials.

## Supplementary Materials

The following supporting information can be downloaded at: https://www.mdpi.com/article/10.3390/ijms26073169/s1.

## Abbreviations

The following abbreviations are used in this manuscript:

Aβ	Amyloid beta
AD	Alzheimer’s disease
ALT	Alanine Aminotransferase
APP/PS1	Amyloid Precursor Protein/Presenilin 1 (transgenic mice model)
AST	Aspartate Aminotransferase
BUN	Blood Urea Nitrogen
BLFs	Bamboo Leaf Flavonoids
CD	Circadian Disruption
Cr	Creatinine
DAPI	4′,6-diamidino-2-phenylindole (nuclear stain)
DESeq	Differential Expression Sequencing
DMEM	Dulbecco’s Modified Eagle Medium
ECL	Enhanced Chemiluminescence
GFAP	Glial fibrillary acidic protein
GSH	Glutathione
HE	Hematoxylin-Eosin
HIF3α	Hypoxia-inducible factor 3 alpha
IL-1β	Interleukin 1 beta
Iba-1	Ionized Calcium-binding Adapter Molecule 1
MDA	Malondialdehyde
NF-κB	Nuclear Factor kappa-light-chain-enhancer of activated B cells
PC12	Rat adrenal pheochromocytoma cell line
PBS	Phosphate-buffered saline
PMSF	Phenylmethylsulfonyl fluoride
Rab7	Ras-associated binding protein 7
ROS	Reactive oxygen species
siRNA	Small interfering RNA
SPF	Specific pathogen-free
TBST	Tris-buffered saline with Tween
