# Supplementary material for: Bamboo Leaf Flavonoids from Phyllostachys glauca McClure Suppress the Progression of Alzheimer’s Disease Induced by Circadian Rhythm Disruption Through Regulating Hif3α/Rab7/TNFα/IL1β Pathway
Source: Int J Mol Sci. 2025 Mar 29;26(7):3169. doi: 10.3390/ijms26073169 (PMC11989969; doi:10.3390/ijms26073169)
Supplement: Supplementary file 1 [file ijms-26-03169-s001.zip › ijms-3538517-supplementary.pdf]

Abbreviations

Aβ: Amyloid beta

AD: Alzheimer's disease

ALT: Alanine Aminotransferase

APP/PS1: Amyloid Precursor Protein/Presenilin 1 (transgenic mice model)

AST: Aspartate Aminotransferase

BUN: Blood Urea Nitrogen

BLFs: Bamboo Leaf Flavonoids

CD: Circadian Disruption

Cr: Creatinine

DAPI: 4',6-diamidino-2-phenylindole (nuclear stain)

DESeq: Differential Expression Sequencing

DMEM: Dulbecco's Modified Eagle Medium

ECL: Enhanced Chemiluminescence

GFAP: Glial fibrillary acidic protein

GSH: Glutathione

HE: Hematoxylin-Eosin

HIF3α: Hypoxia-inducible factor 3 alpha

IL-1β: Interleukin 1 beta

Iba-1: Ionized Calcium-binding Adapter Molecule 1

MDA: Malondialdehyde

NF-κB: Nuclear Factor kappa-light-chain-enhancer of activated B cells

PC12: Rat adrenal pheochromocytoma cell line

PBS: Phosphate-buffered saline

PMSF: Phenylmethylsulfonyl fluoride

Rab7: Ras-associated binding protein 7

ROS: Reactive oxygen species

siRNA: Small interfering RNA

SPF: Specific pathogen-free

TBST: Tris-buffered saline with Tween

Table S1 Sources of Materials and Reagents

Materials and Reagents	Supplier
Specific pathogen-free (SPF) male C57BL/6N mice and male double transgenic APP/PS1 mice (both aged 8–10 weeks)	Huachuangxinnuo Company (Nanjing, Jiangsu Province, China).
Butylated hydroxytoluene flavonoids (BLFs; purity ≥99.8%)	J&K Scientific (Beijing, China)
Primary antibodies related to GFAP/Iba-1 fluorescent staining, Amyloid-β (Aβ), Hif3α, Rab7, TNFα, and IL-1β , DAPI staining, the corresponding secondary antibodies and beta-Amyloid (1-42)	Abcam (Shanghai, China)
Sodium citrate antigen retrieval solution	Beyotime Biotechnology Co., Ltd.
PC12 cell line	ATCC (Manassas, Virginia, USA)
siRNA sequences	RiboBio Co., Ltd (Guangzhou, Guangdong Province, China)
Lipofectamine 2000	Thermo Fisher Scientific (Waltham, MA, USA)
Hematoxylin-eosin (HE) staining kits, Nissl staining solution, biochemical indicator reagents, DEME,and all Western blot-related solutions	Solarbio Company (Beijing, China)

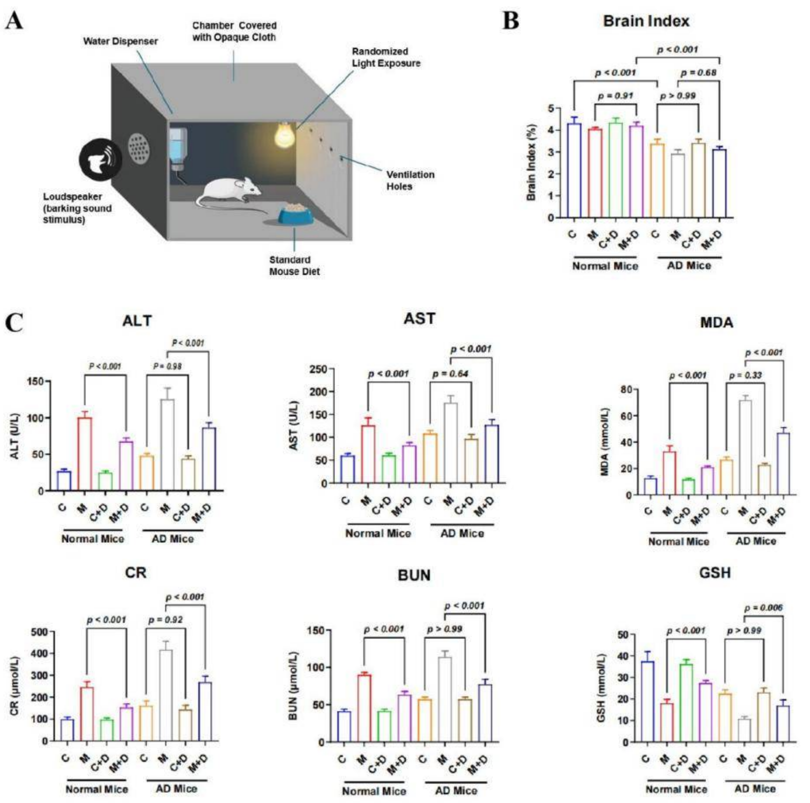

Figure S1. Brain index and biochemical indicators detection. (A) Schematic representation of the animal model used in the study. Mice were housed in a controlled environment with randomized light exposure, loudspeaker stimulation, and other stress-inducing factors to simulate circadian disruption. (B) The Brain Index was calculated as the ratio of brain weight to total body weight, expressed as a percentage, in both normal and AD mice across various treatment groups. (C) Serum biochemical indicators of liver function (ALT and AST), oxidative stress (MDA and GSH), and renal function (BUN and CR) are shown for the different experimental groups. Statistical significance (p-values) is indicated for each comparison. n=5.

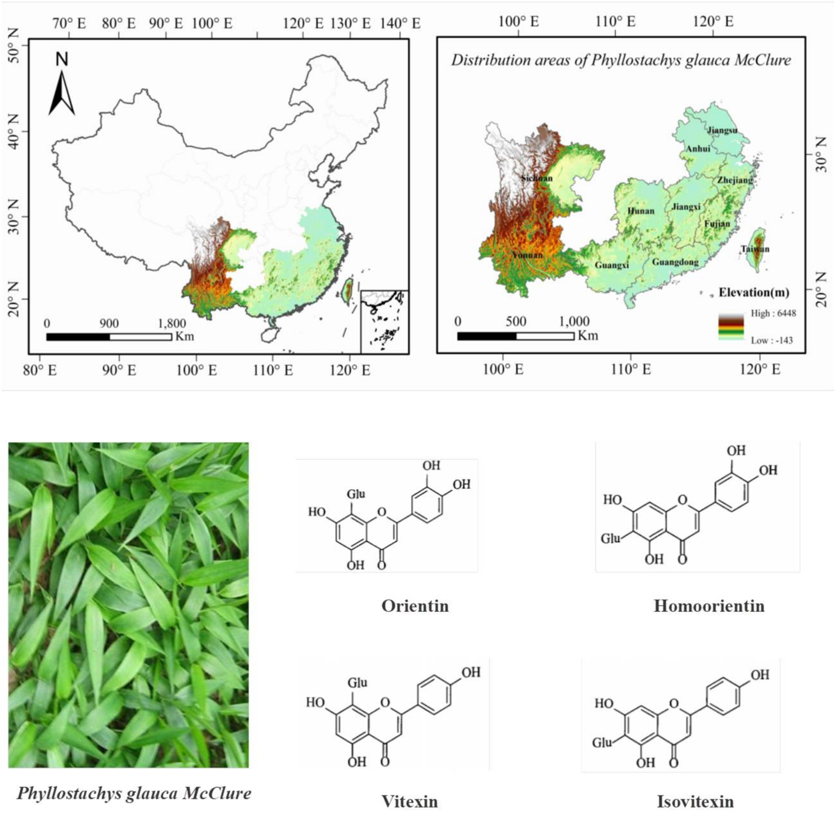

Figure S2. The space distributions of Phyllostachys glauca McClure in P.R. China, and four main flavonoids in Phyllostachys glauca McClure bamboo leaves.
